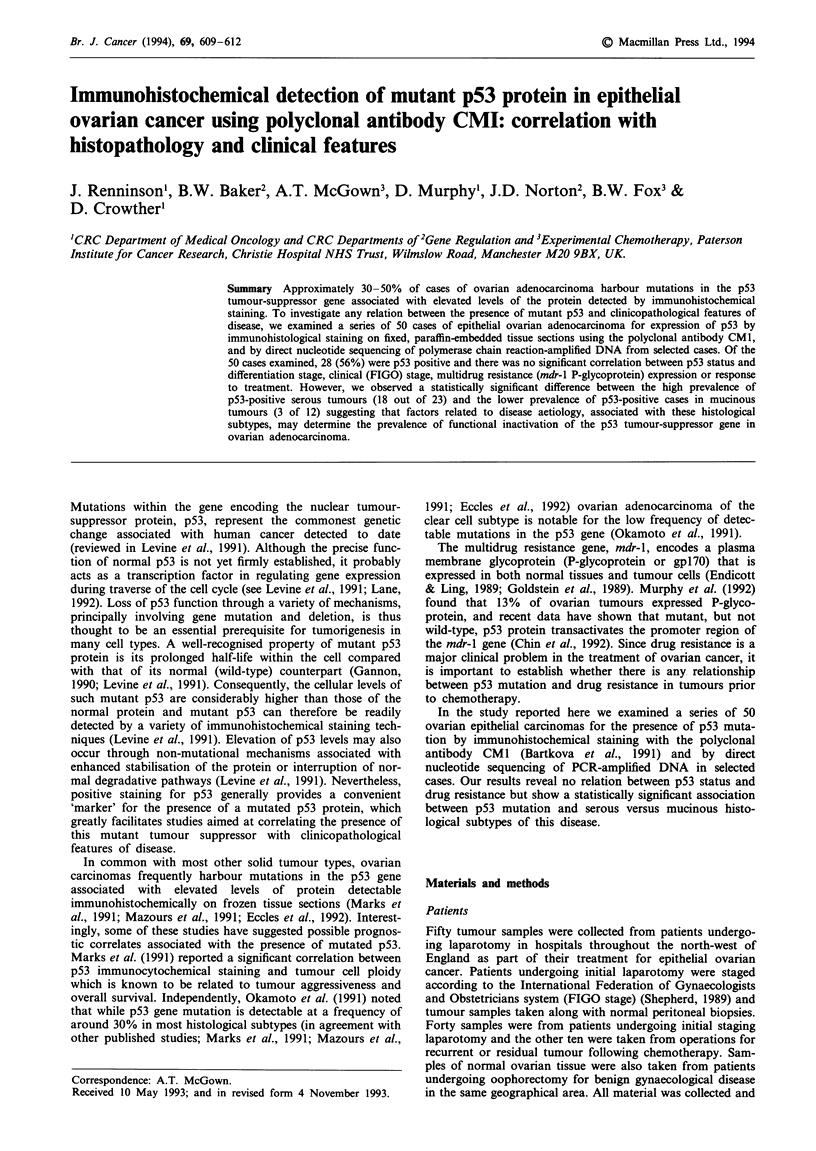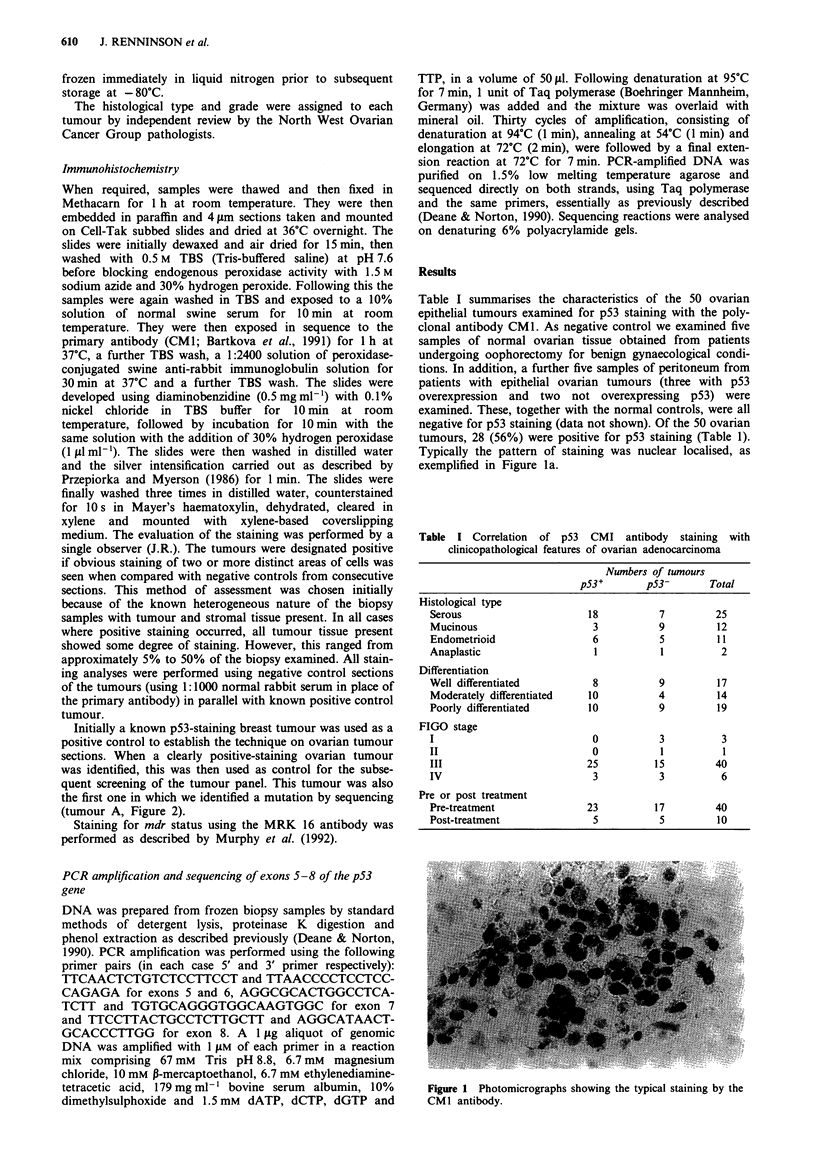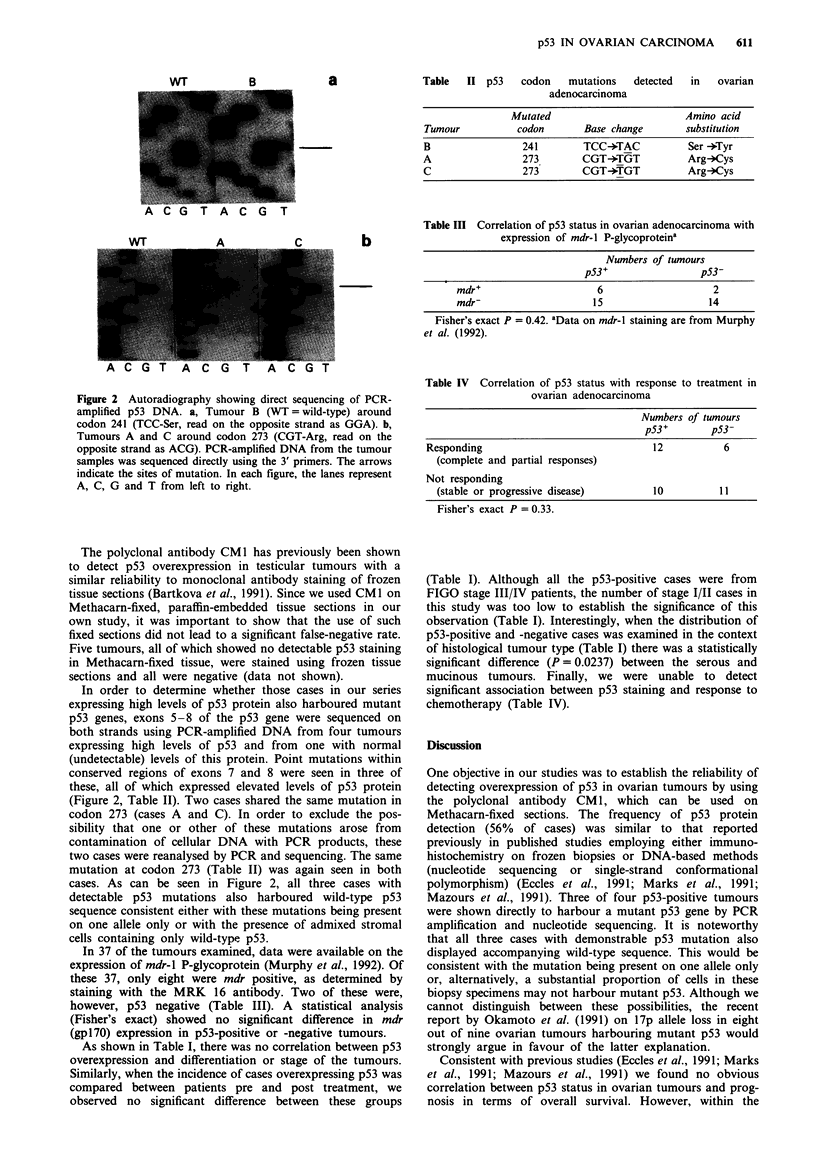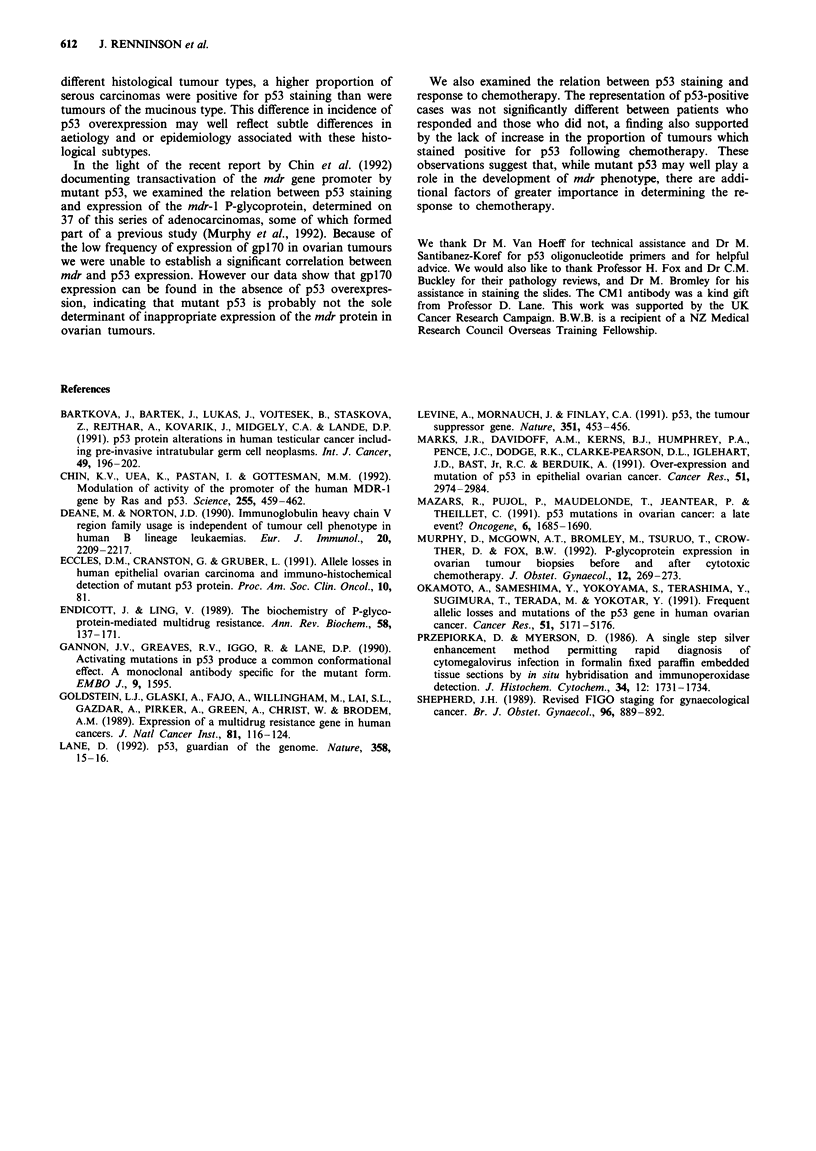# Immunohistochemical detection of mutant p53 protein in epithelial ovarian cancer using polyclonal antibody CMI: correlation with histopathology and clinical features.

**DOI:** 10.1038/bjc.1994.113

**Published:** 1994-03

**Authors:** J. Renninson, B. W. Baker, A. T. McGown, D. Murphy, J. D. Norton, B. W. Fox, D. Crowther

**Affiliations:** CRC Department of Medical Oncology, Paterson Institute for Cancer Research, Christie Hospital NHS Trust, Manchester, UK.

## Abstract

**Images:**


Br. J. Cancer (1994), 69, 609-612 © Macmillan Press Ltd., 1994

### Immunohistochemical detection of mutant p53 protein in epithelial ovarian cancer using polyclonal antibody CMI: correlation with histopathology and clinical features

J. Renninson[1], B.W. Baker[2], A.T. McGown[3], D. Murphy[1], J.D. Norton[2], B.W. Fox[3] & D. Crowther[1]

[1]CRC Department of Medical Oncology and CRC Departments of [2]Gene Regulation and [3]Experimental Chemotherapy, Paterson Institute for Cancer Research, Christie Hospital NHS Trust, Wilmslow Road, Manchester M20 9BX, UK.

**Summary** Approximately 30-50% of cases of ovarian adenocarcinoma harbour mutations in the p53 tumour-suppressor gene associated with elevated levels of the protein detected by immunohistochemical staining. To investigate any relation between the presence of mutant p53 and clinicopathological features of disease, we examined a series of 50 cases of epithelial ovarian adenocarcinoma for expression of p53 by immunohistological staining on fixed, paraffin-embedded tissue sections using the polyclonal antibody CM1, and by direct nucleotide sequencing of polymerase chain reaction-amplified DNA from selected cases. Of the 50 cases examined, 28 (56%) were p53 positive and there was no significant correlation between p53 status and differentiation stage, clinical (FIGO) stage, multidrug resistance (*mdr*-1 P-glycoprotein) expression or response to treatment. However, we observed a statistically significant difference between the high prevalence of p53-positive serous tumours (18 out of 23) and the lower prevalence of p53-positive cases in mucinous tumours (3 of 12) suggesting that factors related to disease aetiology, associated with these histological subtypes, may determine the prevalence of functional inactivation of the p53 tumour-suppressor gene in ovarian adenocarcinoma.

Mutations within the gene encoding the nuclear tumour-suppressor protein, p53, represent the commonest genetic change associated with human cancer detected to date (reviewed in Levine *et al.*, 1991). Although the precise function of normal p53 is not yet firmly established, it probably acts as a transcription factor in regulating gene expression during traverse of the cell cycle (see Levine *et al.*, 1991; Lane, 1992). Loss of p53 function through a variety of mechanisms, principally involving gene mutation and deletion, is thus thought to be an essential prerequisite for tumorigenesis in many cell types. A well-recognised property of mutant p53 protein is its prolonged half-life within the cell compared with that of its normal (wild-type) counterpart (Gannon, 1990; Levine *et al.*, 1991). Consequently, the cellular levels of such mutant p53 are considerably higher than those of the normal protein and mutant p53 can therefore be readily detected by a variety of immunohistochemical staining techniques (Levine *et al.*, 1991). Elevation of p53 levels may also occur through non-mutational mechanisms associated with enhanced stabilisation of the protein or interruption of normal degradative pathways (Levine *et al.*, 1991). Nevertheless, positive staining for p53 generally provides a convenient 'marker' for the presence of a mutated p53 protein, which greatly facilitates studies aimed at correlating the presence of this mutant tumour suppressor with clinicopathological features of disease.

In common with most other solid tumour types, ovarian carcinomas frequently harbour mutations in the p53 gene associated with elevated levels of protein detectable immunohistochemically on frozen tissue sections (Marks *et al.*, 1991; Mazours *et al.*, 1991; Eccles *et al.*, 1992). Interestingly, some of these studies have suggested possible prognostic correlates associated with the presence of mutated p53. Marks *et al.* (1991) reported a significant correlation between p53 immunocytochemical staining and tumour cell ploidy which is known to be related to tumour aggressiveness and overall survival. Independently, Okamoto *et al.* (1991) noted that while p53 gene mutation is detectable at a frequency of around 30% in most histological subtypes (in agreement with other published studies; Marks *et al.*, 1991; Mazours *et al.*, 1991; Eccles *et al.*, 1992) ovarian adenocarcinoma of the clear cell subtype is notable for the low frequency of detectable mutations in the p53 gene (Okamoto *et al.*, 1991).

The multidrug resistance gene, *mdr*-1, encodes a plasma membrane glycoprotein (P-glycoprotein or gp170) that is expressed in both normal tissues and tumour cells (Endicott & Ling, 1989; Goldstein *et al.*, 1989). Murphy *et al.* (1992) found that 13% of ovarian tumours expressed P-glycoprotein, and recent data have shown that mutant, but not wild-type, p53 protein transactivates the promoter region of the *mdr*-1 gene (Chin *et al.*, 1992). Since drug resistance is a major clinical problem in the treatment of ovarian cancer, it is important to establish whether there is any relationship between p53 mutation and drug resistance in tumours prior to chemotherapy.

In the study reported here we examined a series of 50 ovarian epithelial carcinomas for the presence of p53 mutation by immunohistochemical staining with the polyclonal antibody CM1 (Bartkova *et al.*, 1991) and by direct nucleotide sequencing of PCR-amplified DNA in selected cases. Our results reveal no relation between p53 status and drug resistance but show a statistically significant association between p53 mutation and serous versus mucinous histological subtypes of this disease.

### Materials and methods

### Patients

Fifty tumour samples were collected from patients undergoing laparotomy in hospitals throughout the north-west of England as part of their treatment for epithelial ovarian cancer. Patients undergoing initial laparotomy were staged according to the International Federation of Gynaecologists and Obstetricians system (FIGO stage) (Shepherd, 1989) and tumour samples taken along with normal peritoneal biopsies. Forty samples were from patients undergoing initial staging laparotomy and the other ten were taken from operations for recurrent or residual tumour following chemotherapy. Samples of normal ovarian tissue were also taken from patients undergoing oophorectomy for benign gynaecological disease in the same geographical area. All material was collected and

Correspondence: A.T. McGown.
Received 10 May 1993; and in revised form 4 November 1993.

frozen immediately in liquid nitrogen prior to subsequent storage at −80°C.

The histological type and grade were assigned to each tumour by independent review by the North West Ovarian Cancer Group pathologists.

*Immunohistochemistry*

When required, samples were thawed and then fixed in Methacarn for 1 h at room temperature. They were then embedded in paraffin and 4 μm sections taken and mounted on Cell-Tak subbed slides and dried at 36°C overnight. The slides were initially dewaxed and air dried for 15 min, then washed with 0.5 M TBS (Tris-buffered saline) at pH 7.6 before blocking endogenous peroxidase activity with 1.5 M sodium azide and 30% hydrogen peroxide. Following this the samples were again washed in TBS and exposed to a 10% solution of normal swine serum for 10 min at room temperature. They were then exposed in sequence to the primary antibody (CM1; Bartkova et al., 1991) for 1 h at 37°C, a further TBS wash, a 1:2400 solution of peroxidase-conjugated swine anti-rabbit immunoglobulin solution for 30 min at 37°C and a further TBS wash. The slides were developed using diaminobenzidine (0.5 mg $ml^{-1}$) with 0.1% nickel chloride in TBS buffer for 10 min at room temperature, followed by incubation for 10 min with the same solution with the addition of 30% hydrogen peroxidase (1 μl $ml^{-1}$). The slides were then washed in distilled water and the silver intensification carried out as described by Przepiorka and Myerson (1986) for 1 min. The slides were finally washed three times in distilled water, counterstained for 10 s in Mayer's haematoxylin, dehydrated, cleared in xylene and mounted with xylene-based coverslipping medium. The evaluation of the staining was performed by a single observer (J.R.). The tumours were designated positive if obvious staining of two or more distinct areas of cells was seen when compared with negative controls from consecutive sections. This method of assessment was chosen initially because of the known heterogeneous nature of the biopsy samples with tumour and stromal tissue present. In all cases where positive staining occurred, all tumour tissue present showed some degree of staining. However, this ranged from approximately 5% to 50% of the biopsy examined. All staining analyses were performed using negative control sections of the tumours (using 1:1000 normal rabbit serum in place of the primary antibody) in parallel with known positive control tumour.

Initially a known p53-staining breast tumour was used as a positive control to establish the technique on ovarian tumour sections. When a clearly positive-staining ovarian tumour was identified, this was then used as control for the subsequent screening of the tumour panel. This tumour was also the first one in which we identified a mutation by sequencing (tumour A, Figure 2).

Staining for *mdr* status using the MRK 16 antibody was performed as described by Murphy et al. (1992).

*PCR amplification and sequencing of exons 5–8 of the p53 gene*

DNA was prepared from frozen biopsy samples by standard methods of detergent lysis, proteinase K digestion and phenol extraction as described previously (Deane & Norton, 1990). PCR amplification was performed using the following primer pairs (in each case 5′ and 3′ primer respectively): TTCAACTCTGTCTCCTTCCT and TTAACCCCTCCTCCCAGAGA for exons 5 and 6, AGGCGCACTGGCCTCATCTT and TGTGCAGGGTGGCAAGTGGC for exon 7 and TTCCTTACTGCCTCTTGCTT and AGGCATAACTGCACCCTTGG for exon 8. A 1 μg aliquot of genomic DNA was amplified with 1 μM of each primer in a reaction mix comprising 67 mM Tris pH 8.8, 6.7 mM magnesium chloride, 10 mM β-mercaptoethanol, 6.7 mM ethylenediaminetetracetic acid, 179 mg $ml^{-1}$ bovine serum albumin, 10% dimethylsulphoxide and 1.5 mM dATP, dCTP, dGTP and TTP, in a volume of 50 μl. Following denaturation at 95°C for 7 min, 1 unit of Taq polymerase (Boehringer Mannheim, Germany) was added and the mixture was overlaid with mineral oil. Thirty cycles of amplification, consisting of denaturation at 94°C (1 min), annealing at 54°C (1 min) and elongation at 72°C (2 min), were followed by a final extension reaction at 72°C for 7 min. PCR-amplified DNA was purified on 1.5% low melting temperature agarose and sequenced directly on both strands, using Taq polymerase and the same primers, essentially as previously described (Deane & Norton, 1990). Sequencing reactions were analysed on denaturing 6% polyacrylamide gels.

### Results

Table I summarises the characteristics of the 50 ovarian epithelial tumours examined for p53 staining with the polyclonal antibody CM1. As negative control we examined five samples of normal ovarian tissue obtained from patients undergoing oophorectomy for benign gynaecological conditions. In addition, a further five samples of peritoneum from patients with epithelial ovarian tumours (three with p53 overexpression and two not overexpressing p53) were examined. These, together with the normal controls, were all negative for p53 staining (data not shown). Of the 50 ovarian tumours, 28 (56%) were positive for p53 staining (Table 1). Typically the pattern of staining was nuclear localised, as exemplified in Figure 1a.

**Table I** Correlation of p53 CMI antibody staining with clinicopathological features of ovarian adenocarcinoma

| | *Numbers of tumours* | | |
|---|---|---|---|
| | *p53⁺* | *p53⁻* | *Total* |
| Histological type | | | |
| Serous | 18 | 7 | 25 |
| Mucinous | 3 | 9 | 12 |
| Endometrioid | 6 | 5 | 11 |
| Anaplastic | 1 | 1 | 2 |
| Differentiation | | | |
| Well differentiated | 8 | 9 | 17 |
| Moderately differentiated | 10 | 4 | 14 |
| Poorly differentiated | 10 | 9 | 19 |
| FIGO stage | | | |
| I | 0 | 3 | 3 |
| II | 0 | 1 | 1 |
| III | 25 | 15 | 40 |
| IV | 3 | 3 | 6 |
| Pre or post treatment | | | |
| Pre-treatment | 23 | 17 | 40 |
| Post-treatment | 5 | 5 | 10 |

**Figure 1** Photomicrographs showing the typical staining by the CM1 antibody.

**Figure 2** Autoradiography showing direct sequencing of PCR-amplified p53 DNA. **a**, Tumour B (WT = wild-type) around codon 241 (TCC-Ser, read on the opposite strand as GGA). **b**, Tumours A and C around codon 273 (CGT-Arg, read on the opposite strand as ACG). PCR-amplified DNA from the tumour samples was sequenced directly using the 3′ primers. The arrows indicate the sites of mutation. In each figure, the lanes represent A, C, G and T from left to right.

**Table II** p53 codon mutations detected in ovarian adenocarcinoma

| *Tumour* | *Mutated codon* | *Base change* | *Amino acid substitution* |
|---|---|---|---|
| B | 241 | TCC→TAC | Ser →Tyr |
| A | 273 | CGT→TGT | Arg→Cys |
| C | 273 | CGT→TGT | Arg→Cys |

**Table III** Correlation of p53 status in ovarian adenocarcinoma with expression of *mdr*-1 P-glycoprotein[a]

| | *Numbers of tumours* | |
|---|---|---|
| | *p53*$^+$ | *p53*$^-$ |
| *mdr*$^+$ | 6 | 2 |
| *mdr*$^-$ | 15 | 14 |

Fisher's exact $P = 0.42$. [a]Data on *mdr*-1 staining are from Murphy *et al.* (1992).

**Table IV** Correlation of p53 status with response to treatment in ovarian adenocarcinoma

| | *Numbers of tumours* | |
|---|---|---|
| | *p53*$^+$ | *p53*$^-$ |
| Responding (complete and partial responses) | 12 | 6 |
| Not responding (stable or progressive disease) | 10 | 11 |

Fisher's exact $P = 0.33$.

The polyclonal antibody CM1 has previously been shown to detect p53 overexpression in testicular tumours with a similar reliability to monoclonal antibody staining of frozen tissue sections (Bartkova *et al.*, 1991). Since we used CM1 on Methacarn-fixed, paraffin-embedded tissue sections in our own study, it was important to show that the use of such fixed sections did not lead to a significant false-negative rate. Five tumours, all of which showed no detectable p53 staining in Methacarn-fixed tissue, were stained using frozen tissue sections and all were negative (data not shown).

In order to determine whether those cases in our series expressing high levels of p53 protein also harboured mutant p53 genes, exons 5–8 of the p53 gene were sequenced on both strands using PCR-amplified DNA from four tumours expressing high levels of p53 and from one with normal (undetectable) levels of this protein. Point mutations within conserved regions of exons 7 and 8 were seen in three of these, all of which expressed elevated levels of p53 protein (Figure 2, Table II). Two cases shared the same mutation in codon 273 (cases A and C). In order to exclude the possibility that one or other of these mutations arose from contamination of cellular DNA with PCR products, these two cases were reanalysed by PCR and sequencing. The same mutation at codon 273 (Table II) was again seen in both cases. As can be seen in Figure 2, all three cases with detectable p53 mutations also harboured wild-type p53 sequence consistent either with these mutations being present on one allele only or with the presence of admixed stromal cells containing only wild-type p53.

In 37 of the tumours examined, data were available on the expression of *mdr*-1 P-glycoprotein (Murphy *et al.*, 1992). Of these 37, only eight were *mdr* positive, as determined by staining with the MRK 16 antibody. Two of these were, however, p53 negative (Table III). A statistical analysis (Fisher's exact) showed no significant difference in *mdr* (gp170) expression in p53-positive or -negative tumours.

As shown in Table I, there was no correlation between p53 overexpression and differentiation or stage of the tumours. Similarly, when the incidence of cases overexpressing p53 was compared between patients pre and post treatment, we observed no significant difference between these groups (Table I). Although all the p53-positive cases were from FIGO stage III/IV patients, the number of stage I/II cases in this study was too low to establish the significance of this observation (Table I). Interestingly, when the distribution of p53-positive and -negative cases was examined in the context of histological tumour type (Table I) there was a statistically significant difference ($P = 0.0237$) between the serous and mucinous tumours. Finally, we were unable to detect significant association between p53 staining and response to chemotherapy (Table IV).

### Discussion

One objective in our studies was to establish the reliability of detecting overexpression of p53 in ovarian tumours by using the polyclonal antibody CM1, which can be used on Methacarn-fixed sections. The frequency of p53 protein detection (56% of cases) was similar to that reported previously in published studies employing either immunohistochemistry on frozen biopsies or DNA-based methods (nucleotide sequencing or single-strand conformational polymorphism) (Eccles *et al.*, 1991; Marks *et al.*, 1991; Mazours *et al.*, 1991). Three of four p53-positive tumours were shown directly to harbour a mutant p53 gene by PCR amplification and nucleotide sequencing. It is noteworthy that all three cases with demonstrable p53 mutation also displayed accompanying wild-type sequence. This would be consistent with the mutation being present on one allele only or, alternatively, a substantial proportion of cells in these biopsy specimens may not harbour mutant p53. Although we cannot distinguish between these possibilities, the recent report by Okamoto *et al.* (1991) on 17p allele loss in eight out of nine ovarian tumours harbouring mutant p53 would strongly argue in favour of the latter explanation.

Consistent with previous studies (Eccles *et al.*, 1991; Marks *et al.*, 1991; Mazours *et al.*, 1991) we found no obvious correlation between p53 status in ovarian tumours and prognosis in terms of overall survival. However, within the different histological tumour types, a higher proportion of serous carcinomas were positive for p53 staining than were tumours of the mucinous type. This difference in incidence of p53 overexpression may well reflect subtle differences in aetiology and or epidemiology associated with these histological subtypes.

In the light of the recent report by Chin *et al.* (1992) documenting transactivation of the *mdr* gene promoter by mutant p53, we examined the relation between p53 staining and expression of the *mdr*-1 P-glycoprotein, determined on 37 of this series of adenocarcinomas, some of which formed part of a previous study (Murphy *et al.*, 1992). Because of the low frequency of expression of gp170 in ovarian tumours we were unable to establish a significant correlation between *mdr* and p53 expression. However our data show that gp170 expression can be found in the absence of p53 overexpression, indicating that mutant p53 is probably not the sole determinant of inappropriate expression of the *mdr* protein in ovarian tumours.

We also examined the relation between p53 staining and response to chemotherapy. The representation of p53-positive cases was not significantly different between patients who responded and those who did not, a finding also supported by the lack of increase in the proportion of tumours which stained positive for p53 following chemotherapy. These observations suggest that, while mutant p53 may well play a role in the development of *mdr* phenotype, there are additional factors of greater importance in determining the response to chemotherapy.

We thank Dr M. Van Hoeff for technical assistance and Dr M. Santibanez-Koref for p53 oligonucleotide primers and for helpful advice. We would also like to thank Professor H. Fox and Dr C.M. Buckley for their pathology reviews, and Dr M. Bromley for his assistance in staining the slides. The CM1 antibody was a kind gift from Professor D. Lane. This work was supported by the UK Cancer Research Campaign. B.W.B. is a recipient of a NZ Medical Research Council Overseas Training Fellowship.

### References

BARTKOVA, J., BARTEK, J., LUKAS, J., VOJTESEK, B., STASKOVA, Z., REJTHAR, A., KOVARIK, J., MIDGELY, C.A. & LANDE, D.P. (1991). p53 protein alterations in human testicular cancer including pre-invasive intratubular germ cell neoplasms. *Int. J. Cancer*, **49**, 196–202.

CHIN, K.V., UEA, K., PASTAN, I. & GOTTESMAN, M.M. (1992). Modulation of activity of the promoter of the human MDR-1 gene by Ras and p53. *Science*, **255**, 459–462.

DEANE, M. & NORTON, J.D. (1990). Immunoglobulin heavy chain V region family usage is independent of tumour cell phenotype in human B lineage leukaemias. *Eur. J. Immunol.*, **20**, 2209–2217.

ECCLES, D.M., CRANSTON, G. & GRUBER, L. (1991). Allele losses in human epithelial ovarian carcinoma and immuno-histochemical detection of mutant p53 protein. *Proc. Am. Soc. Clin. Oncol.*, **10**, 81.

ENDICOTT, J. & LING, V. (1989). The biochemistry of P-glycoprotein-mediated multidrug resistance. *Ann. Rev. Biochem.*, **58**, 137–171.

GANNON, J.V., GREAVES, R.V., IGGO, R. & LANE, D.P. (1990). Activating mutations in p53 produce a common conformational effect. A monoclonal antibody specific for the mutant form. *EMBO J.*, **9**, 1595.

GOLDSTEIN, L.J., GLASKI, A., FAJO, A., WILLINGHAM, M., LAI, S.L., GAZDAR, A., PIRKER, A., GREEN, A., CHRIST, W. & BRODEM, A.M. (1989). Expression of a multidrug resistance gene in human cancers. *J. Natl Cancer Inst.*, **81**, 116–124.

LANE, D. (1992). p53, guardian of the genome. *Nature*, **358**, 15–16.

LEVINE, A., MORNAUCH, J. & FINLAY, C.A. (1991). p53, the tumour suppressor gene. *Nature*, **351**, 453–456.

MARKS, J.R., DAVIDOFF, A.M., KERNS, B.J., HUMPHREY, P.A., PENCE, J.C., DODGE, R.K., CLARKE-PEARSON, D.L., IGLEHART, J.D., BAST, Jr, R.C. & BERDUIK, A. (1991). Over-expression and mutation of p53 in epithelial ovarian cancer. *Cancer Res.*, **51**, 2974–2984.

MAZARS, R., PUJOL, P., MAUDELONDE, T., JEANTEAR, P. & THEILLET, C. (1991). p53 mutations in ovarian cancer: a late event? *Oncogene*, **6**, 1685–1690.

MURPHY, D., MCGOWN, A.T., BROMLEY, M., TSURUO, T., CROWTHER, D. & FOX, B.W. (1992). P-glycoprotein expression in ovarian tumour biopsies before and after cytotoxic chemotherapy. *J. Obstet. Gynaecol.*, **12**, 269–273.

OKAMOTO, A., SAMESHIMA, Y., YOKOYAMA, S., TERASHIMA, Y., SUGIMURA, T., TERADA, M. & YOKOTAR, Y. (1991). Frequent allelic losses and mutations of the p53 gene in human ovarian cancer. *Cancer Res.*, **51**, 5171–5176.

PRZEPIORKA, D. & MYERSON, D. (1986). A single step silver enhancement method permitting rapid diagnosis of cytomegalovirus infection in formalin fixed paraffin embedded tissue sections by *in situ* hybridisation and immunoperoxidase detection. *J. Histochem. Cytochem.*, **34**, 12: 1731–1734.

SHEPHERD, J.H. (1989). Revised FIGO staging for gynaecological cancer. *Br. J. Obstet. Gynaecol.*, **96**, 889–892.